# Clinical Impact of CTLA4 and LAG3 Single‐Nucleotide Polymorphisms in Multiple Myeloma Patients Treated With BCMA CAR T‐Cell Therapy

**DOI:** 10.1002/hon.70267

**Published:** 2026-09-28

**Authors:** Elisa Suter, Martina Bertschinger, Inna Shaforostova, Marie‐Noelle Kronig, Henning Nilius, Ulrike Bacher, Katja Seipel, Thomas Pabst

**Affiliations:** ^1^ Department of Medical Oncology Inselspital Bern University Hospital University of Bern Bern Switzerland; ^2^ Department of Clinical Chemistry Inselspital Bern University Hospital Bern Switzerland; ^3^ Department of Hematology Inselspital Bern University Hospital Bern Switzerland; ^4^ Department for Biomedical Research (DBMR) University of Bern Bern Switzerland

## Abstract

Chimeric antigen receptor (CAR) T‐cell therapy targeting B‐cell maturation antigen (BCMA) is a highly effective treatment option for patients with relapsed refractory multiple myeloma (RRMM). However, reliable biomarkers predicting long‐term treatment response remain elusive. Germline single‐nucleotide polymorphisms (SNPs) in immune checkpoint regulators, including cytotoxic T‐lymphocyte–associated protein 4 (CTLA4) and lymphocyte activation gene 3 (LAG3) may affect CAR T‐cell functionality and clinical efficacy. We conducted a retrospective analysis of 87 patients with RRMM treated with BCMA‐directed CAR T‐cells at a single academic center between May 2021 and September 2025, evaluating the impact of CTLA4 rs231775 and LAG3 rs870849 on relapse, progression‐free survival (PFS), and overall survival (OS). The minor allele rs231775 of CTLA4 was present in 48%, while the minor allele of LAG3 rs870849 was observed in 87% of patients. Carriers of the CTLA4 rs231775 minor allele demonstrated lower relapse (40 vs. 53%) and mortality rates (31 vs. 44%) compared with major allele homozygotes, accompanied by significantly prolonged PFS and OS. Likewise, patients homozygous for the LAG3 rs870849 minor allele experienced reduced relapse (36 vs. 52%) and mortality rates (28 vs. 42%), significantly longer PFS, and a trend toward improved OS relative to carriers of the major allele. In conclusion, in our study, the minor alleles of CTLA4 rs231775 and LAG3 rs870849 were associated with superior clinical outcomes following BCMA‐directed CAR T‐cell therapy in RRMM patients. These findings suggest to further evaluate whether immune checkpoint SNPs could be biomarkers predicting response to BCMA‐directed CAR T‐cell therapy in MM which needs prospective studies and validation.

## 1 Introduction

Chimeric antigen receptor (CAR) T‐cell therapy has transformed the therapeutic landscape of hematological malignancies, achieving unprecedented efficacy in patients with relapsed refractory multiple myeloma (RRMM). Clinical trials and real‐world data consistently report primary overall response rates (ORR) exceeding 80%, enabling durable progression‐free survival (PFS) and overall survival (OS) in heavily pretreated MM patients [1, 2, 3]. CARs are genetically engineered fusion receptors that redirect autologous T‐cells toward tumor‐associated antigens by combining an extracellular antigen‐recognition with intracellular T‐cell activation and co‐stimulatory signaling domains. This genetic modification enables patient‐derived T‐cells to efficiently and specifically eliminate malignant cells. In MM, B‐cell maturation antigen (BCMA) has emerged as the most clinically successful CAR target, owing to its high‐level expression on malignant plasma cells, while being restricted to the plasma cell lineage and therefore results in minimal on‐target off‐tumor side effects [4, 5, 6].

Despite these remarkable outcomes, MM patients may ultimately relapse after anti‐BCMA CAR T‐cell therapy, with a reported pooled relapse rate of 38% among responders. Several mechanisms leading to therapy resistance have been identified, including antigen escape resulting from BCMA down‐regulation or loss, limited CAR T‐cell persistence, immunosuppressive tumor microenvironment and host‐specific genetic factors, like various single nucleotide polymorphisms (SNPs) [4, 5, 6, 7, 8, 9]. Especially immune checkpoint regulators, like cytotoxic T‐lymphocyte–associated protein 4 (CTLA4) and lymphocyte activation gene 3 (LAG3) SNPs, are presumed to influence clinical outcomes in patients treated with CAR T‐cell therapies [10, 11].

CTLA‐4 is normally expressed on T‐cells, where it regulates the T‐cell activation through counteracting with CD28, a co‐stimulatory receptor of T‐cells [12]. Thereby, CTLA‐4 reduces the activity of antigen presenting cells that stimulate T‐cells via CD28 and limits physiologically autoreactivity. Therapeutic blockade of CTLA‐4 with monoclonal antibodies enhances antitumor immune responses and has become an established treatment strategy in multiple solid malignancies, including melanoma and lung cancer [13, 14]. Several CTLA4 germline genetic variants, such as rs231775, have been associated with autoimmune disease and cancer risk. The rs231775 variant, located within the coding region, results in a threonine‐to‐alanine substitution in the N‐terminal signal peptide. Homozygosity for the minor allele (A17hom) has been associated with impaired glycosylation, reduced surface expression of CTLA‐4, and altered immune regulation [11, 15]. An association between CTLA4 minor allele rs231775 and favorable treatment outcome was shown in diffuse large B‐cell lymphoma (DLBCL) patients treated with CAR T‐cell therapy [11].

Comparable to CTLA‐4, LAG‐3 plays a regulatory role in immunity as an inhibitory checkpoint molecule [16]. Encoded on chromosome 12, LAG3 exhibits multiple genetic variants with potential functional relevance. The LAG3 rs870849 entails the substitution of a nonpolar isoleucine for an uncharged polar threonine in the transmembrane domain (T455hom when homozygous). This change may influence protein function, protein stability, cellular localization, and downstream signaling. This variant has been associated with altered T‐cell cytokine production and enhanced antitumor immunity. Favorable clinical outcomes have been reported in LAG3 rs870849 carriers among patients with DLBCL receiving CAR T‐cell therapy [16, 17, 18, 19]. Moreover, LAG3 rs870849 may affect survival and treatment outcome after autologous stem cell transplantation in MM patients with favorable outcomes in rs870849 carriers [20].

Given the critical role of immune checkpoint signaling molecules in T‐cell function and the emerging evidence of different CTLA4 and LAG3 germline variants to treatment outcomes in lymphoma, their relevance in the management of MM patients warrants further investigation. In this retrospective study, we investigated the prevalence of CTLA4 rs231775 and LAG3 rs870849 allele and the correlation to clinical outcomes and complications in RRMM patients treated with anti‐BCMA CAR T‐cell therapy.

## 2 Materials and Methods

### 2.1 Patients

This retrospective single‐center study at the Inselspital, University Hospital of Bern, Switzerland, included 87 RRMM patients, who received a BCMA‐directed CAR T‐cell therapy between May 2021 and September 2025 with idecabtagen vicleucel (ide‐cel, Abecma) or ciltacabtagene autoleucel (cilta‐cel, Carvykti) following standard lymphodepleting therapy. The study was conducted according to the guidelines of the Declaration of Helsinki and approved by decisions of the local ethics committee of Bern, Switzerland (decision number 2025–00853, date of approval April 24, 2025). Data cut‐off was December 1, 2025.

### 2.2 Study Endpoints

The primary endpoint was clinical outcome including relapse rate, PFS and OS in patients with and without CTLA4 rs231775 and LAG3 rs870849. As secondary endpoints the prevalence of these alleles, as well as the correlations with remission stages, CAR T‐cell persistence, and treatment‐related toxicities were assessed. Cytokine release syndrome (CRS) and immune‐effector cell‐associated neurotoxicity syndrome (ICANS) were assessed according to the consensus grading criteria of the American Society for Transplantation and Cellular Therapy.

### 2.3 CTLA‐4 and LAG‐3 Gene Analysis

The CTLA4 and LAG3 genes were analyzed by Sanger sequencing as previously described [11, 17].

### 2.4 Statistical Analysis

PFS was defined as the time from CAR T‐cell infusion to disease recurrence, death or last follow up. OS was calculated from the date of CAR T‐cell infusion to death from any cause. Categorical variables were assessed using Fisher's exact test, whereas non‐parametric data were analyzed using the Mann‐Whitney test. Survival differences between groups were evaluated by the log‐rank test. Prognostic factors were examined using multivariable Cox proportional hazards models. Assumption of proportional hazards was tested with the Schoenfeld residuals test and a complete case analysis was performed [21]. Statistical analysis and figures were made with GraphPad Prism 10 software.

## 3 Results

### 3.1 Patient Cohort

This cohort comprised 87 patients with RRMM, who received BCMA‐directed CAR T‐cell therapy (Table 1). The cohort showed a male predominance (70%). The median age at initial diagnosis was 58 years. According to the International Staging System (ISS), 24 patients (30%) had stage I, 36 (45%) stage II, and 20 (25%) stage III MM. All patients had received at least one prior line of chemotherapy as well as standard lymphodepleting chemotherapy before CAR T‐cell infusion. Most patients (76%) had also undergone autologous hematopoietic stem cell transplantation prior to CAR T‐cell therapy.

**TABLE 1:** Baseline clinical parameters by CTLA4 and LAG3 genotype.

Parameter	All patients	CTLA4 T17hom	CTLA4 A17hom and T17Ahet	*p*‐value	LAG3T455hom	LAG3 I455hom and I455Thet	*p*‐value
Patients, *n* (%)	87 (100)	45 (52)	42 (48)		25 (29)	62 (72)	
Male to female (ratio)	2.3 (61:26)	2 (30:15)	2.8 (31:11)	0.49^a^	2.6 (18:7)	2.3 (43:19)	0.99^a^
Age at ID, years, median (range)	58 (33–78)	58 (33–73)	58 (43–78)	0.78^b^	58 (33–76)	59 (39–78)	0.76^b^
Initial ISS stage, *n* (%)							
I	24 (30)	8 (19)	16 (43)	0.29^b^	9 (38)	15 (27)	0.38^b^
II	36 (45)	26 (60)	10 (27)		10 (42)	26 (46)	
III	20 (25)	9 (21)	11 (30)		5 (21)	15 (27)	
High‐risk genetic, *n* (%)	21 (33)	14 (40)	7 (25)	0.28^a^	6 (29)	15 (36)	0.78^a^
(Immuno‐)Chemo‐tx lines, *n* (%)							
1–3	40 (46)	18 (40)	22 (52)	0.29^a^	11 (44)	29 (47)	0.99^a^
> 3	47 (54)	27 (60)	20 (48)		14 (56)	33 (53)	
Radiotherapy, *n* (%)	41 (47)	17 (38)	24 (57)	0.09^a^	16 (64)	25 (40)	0.06^a^
Autologous SCT, *n* (%)	66 (76)	34 (76)	32 (76)	0.99^a^	19 (76)	47 (76)	0.99^a^
Allogeneic SCT, *n* (%)	2 (2)	1 (2)	1 (2)	0.99^a^	1 (4)	1 (2)	0.49^a^

Abbreviations: chemo‐tx, chemotherapy; ID, initial diagnosis; ISS, international staging system; SCT, stem cell transplantation.

^a^ Fisher's exact test.

^b^ Mann‐Whitney test.

### 3.2 Prevalence of the CTLA4 rs231775 Allele

Sequencing of the CTLA4 gene exon 1 was performed on peripheral blood samples from 87 patients undergoing CAR T‐cell therapy at Inselspital Bern (Figure 1A). 45 of 87 patients (52%) were homozygous for the major allele encoding T17 (T17hom). 34 patients (39%) were heterozygous for the SNP rs231775 (T17Ahet) and 8 patients (9%) carried two minor alleles (A17hom). These genotype distributions correspond to a minor‐allele frequency (MAF) of 0.29. According to the Allele Frequency Aggregator project (ALFA), rs231775 shows a MAF of 0.37 in the European population (ALFA sample size 509,524, https://www.ncbi.nlm.nih.gov/snp, accessed on November 18, 2025).

**FIGURE 1:**
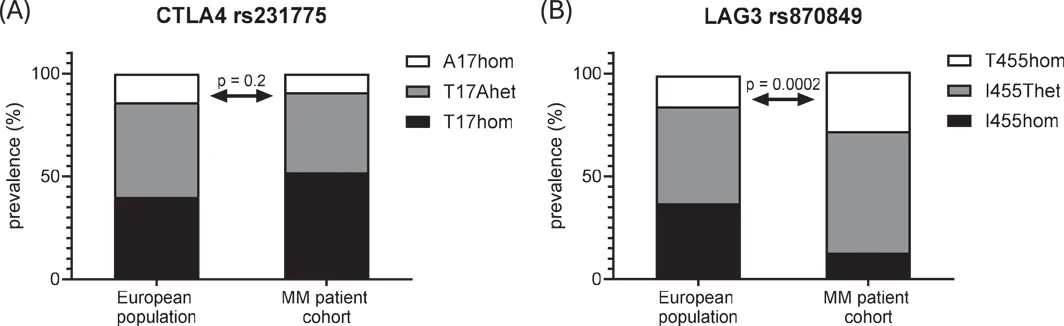
Prevalence of the CTLA4 rs231775 and the LAG3 rs870849 allele in the European population and in the multiple myeloma (MM) patient cohort. Distribution of the (A) CTLA4 rs231775 allele and (B) LAG3 rs870849 allele.

### 3.3 Prevalence of the LAG3 rs870849 Allele

Sequencing of the LAG3 gene exon 7 was conducted in the same cohort of 87 patients (Figure 1B). 11 of 87 patients (13%) carried the homozygous major allele (I455hom), 51 patients (59%) were heterozygous for the SNP rs870849 (I455Thet), and 25 patients (29%) were homozygous for the minor allele (T455hom). Based on this distribution, the MAF of the LAG3 variant in our cohort was 0.58, which is substantially higher than the ALFA‐reported MAF of 0.39 (ALFA sample size 588,090, https://www.ncbi.nlm.nih.gov/snp, accessed on November 18, 2025).

### 3.4 Baseline Clinical Characteristics

Baseline clinical characteristics were evaluated for the total patient cohort as well as for the CTLA4 and LAG3 genetic subgroups (Table 1). Patients harboring either the CTLA4 homozygous (A17hom) or heterozygous (T17Ahet) substitution of threonine to alanine in the N‐terminal signal peptide were compared with those carrying two major alleles (T17hom). The median age at initial diagnosis was 58 years in both groups. High‐risk genetic characteristics were more commonly observed among patients in the T17hom cohort than among those in the A17hom and T17Ahet cohort (40 vs. 25%, *p = 0.28*). Patients in the A17hom and T17Ahet subgroup demonstrated a trend toward receiving more frequently radiotherapy (57 vs. 38%, *p* = 0.09) but received fewer previous lines of (immuno‐) chemotherapy. The distribution of previous autologous and allogeneic hematopoietic stem cell transplantation did not differ between the groups.

For the LAG3 gene, patients carrying the homozygous substitution of the nonpolar isoleucine for an uncharged polar threonine in the transmembrane domain (T455hom) were compared with those patients who were heterozygous (I455Thet) or homozygous for the major allele (I455hom). The median age in the I455hom and I455Thet subgroup was 59 and 58 years in the T455hom subgroup (*p* = 0.76). Additionally, a higher proportion of patients in the I455hom and I455Thet group had advanced ISS stages and high‐risk genetic features (36 vs. 29%, *p* = 0.78) at baseline and less radiotherapy pretreatment (40 vs. 64%, *p* = 0.06). The number of previous (immuno‐)chemotherapy lines administered and the utilization of autologous and allogeneic hematopoietic stem cell transplantation were similar across the different LAG3 subgroups.

### 3.5 Clinical Characteristics and CAR T‐Cell Therapy

Clinical characteristics and CAR T‐cell treatment details showed minimal variation across the genetic subgroups (Table 2). Patients carrying the CTLA4 A17hom or T17Ahet genotype demonstrated a numerically shorter time interval from initial diagnosis to CAR T‐cell infusion (68 vs. 76 days, *p* = 0.89) and received bridging radiotherapy less frequently (7 vs. 13%, *p* = 0.49) compared to the T17hom patients. The utilization of bridging chemotherapy was similar between those groups. The median age at CAR T‐cell infusion was 67 years in both genotype groups. A total of 39 patients (45%) were in progressive disease at the time of CAR T‐cell infusion. Pretreatment lactate dehydrogenase (LDH) levels showed no significant differences between CTLA4 genotype subgroups. Regarding CAR T‐cell product type, the distribution was balanced between the cohorts, with approximately 60% of patients receiving ide‐cel and 40% receiving cilta‐cel (*p* = 0.83).

**TABLE 2:** Clinical characteristics and details of CAR T‐cell treatment by CTLA4 and LAG3 genotype.

Parameter	All patients	CTLA4 T17hom	CTLA4 A17hom and T17Ahet	*p*‐value	LAG3 T455hom	LAG3 I455hom and I455Thet	*p*‐value
Patients, *n* (%)	87 (100)	45 (52)	42 (48)		25 (29)	62 (72)	
Age at CAR *T*, years, median (range)	67 (42–84)	67 (42–84)	67 (50–82)	0.54^b^	66 (42–82)	67 (43–84)	0.99^b^
Time ID to CAR *T*, months, median (range)	73.5 (8–349)	76 (14–349)	68 (8–201)	0.89^b^	79 (8–204)	69 (14–349)	0.43^b^
Time apheresis to CAR *T*, days, median (range)	56 (11–200)	56 (11–200)	56 (42–187)	0.77^b^	54 (42–187)	56 (11–200)	0.85^b^
Bridging chemo‐tx, *n* (%)	48 (55)	26 (58)	22 (52)	0.67^a^	10 (40)	38 (61)	0.10^a^
Bridging radio‐tx, *n* (%)	9 (10)	6 (13)	3 (7)	0.49^a^	2 (8)	7 (11)	0.99^a^
Remission status pre CAR *T*, *n* (%)							
sCR	1 (1)	0 (0)	1 (2)	0.77^b^	1 (4)	0 (0)	0.64^b^
CR	6 (7)	4 (9)	2 (5)		3 (12)	3 (5)	
VGPR	4 (5)	3 (7)	1 (2)		1 (4)	3 (5)	
PR	17 (20)	9 (20)	8 (19)		2 (8)	15 (24)	
SD	20 (23)	8 (18)	12 (29)		8 (32)	13 (21)	
PD	39 (45)	21 (47)	18 (43)		10 (40)	28 (45)	
LDH pre CAR *T*, U/L, median (range)	195 (77–1716)	201 (114–1716)	194.5 (77–1077)	0.48^b^	181 (142–1716)	206.5 (77–1201)	0.13^b^
Elevated LDH (> 250 U/L), *n* (%)	18 (21)	11 (24)	7 (17)	0.43^a^	4 (16)	14 (23)	0.57^a^
Lymphodepleting chemo‐tx, *n* (%)	87 (100)	45 (100)	42 (100)	0.99^a^	25 (100)	62 (100)	0.99^a^
CAR *T* product, *n* (%)							
Ide‐cel	52 (60)	26 (58)	26 (62)	0.83^a^	16 (64)	36 (58)	0.64^a^
Cilta‐cel	35 (40)	19 (42)	16 (38)		9 (36)	26 (42)	

Abbreviations: CAR T, chimeric antigen receptor T‐cell therapy; chemo‐tx, chemotherapy; radio‐tx, radiotherapy; sCR, stringent complete response; CR, complete response; VGPR, very good partial response; PR, partial response; SD, stable disease; PD, progressive disease; LDH, lactate dehydrogenase.

^a^ Fisher's exact test.

^b^ Mann‐Whitney test.

Patients within the LAG3 I455hom and I455Thet group had a numerically shorter interval between initial diagnosis and CAR T‐cell infusion compared to the T455hom subgroup (69 vs. 79 days, *p* = 0.43). The median age at CAR T‐cell therapy was 66 years in the T455hom group and 67 years in the I455hom and I455Thet group (*p* = 0.99). Both bridging chemotherapy (40 vs. 61%, *p* = 0.10) and radiotherapy (8 vs. 11%, *p* = 0.99) were less frequently administered in T455hom patients. Remission status at the time of CAR T‐cell therapy did not differ significantly between LAG3 genotypes. Pretreatment LDH levels tended to be lower in the T455hom cohort (181 vs. 206.5 U/L, *p* = 0.13), accompanied by a reduced proportion of values exceeding the upper limit of normal (16 vs. 23%, *p* = 0.57). In both genotype groups, ide‐cel (T455hom patients 64 vs. I455hom and I455Thet patients 58%, *p* = 0.64) was more commonly used than cilta‐cel.

### 3.6 Treatment Outcome Correlation With CTLA4 Germline Variants, Univariate Analysis

Clinical outcomes and complications following CAR T‐cell therapy were evaluated in 87 patients according to their CTLA4 genotype (Table 3). CRS occurred in a total of 75 patients (86%) at a median onset one day after CAR T‐cell infusion, with a higher incidence in the A17hom and T17Ahet group compared to the T17hom group (90 vs. 82%, *p* = 0.19). Patients from the A17hom and T17Ahet group developed ICANS more frequently (12 vs. 4%, *p* = 0.21). In accordance with these results, the serum peaks of interleukin 6 (1362 vs. 938 pg/mL, *p* = 0.13) and ferritin (1311 vs. 903 μg/L, *p* = 0.33) were higher in the A17hom and T17Ahet group. This group required more tocilizumab administration than the T455hom patient group (90 vs. 80%, *p* = 0.23). Rates of intensive care unit admission and hospitalization duration were comparable between the two cohorts. CAR T‐cell expansion was estimated by measuring cfDNA, serving as indirect surrogate of CAR T‐cell kinetics. The persistence at 6 months did not differ significantly by genotype.

**TABLE 3:** Clinical outcomes after CAR T‐cell treatment by CTLA4 and LAG3 genotype.

Parameter	All patients	CTLA4 T17hom	CTLA4 A17hom&T17Ahet	*p*‐value	LAG3 T455hom	LAG3 I455hom&I455Thet	*p*‐value
Patients *n* (%)	87 (100)	45 (52)	42 (48)		25 (29)	62 (72)	
CRS, *n* (%)							
Total	75 (86)	37 (82)	38 (90)	0.19^b^	20 (80)	55 (89)	0.024^b^
Grade 1	62 (71)	32 (71)	30 (71)		20 (80)	42 (68)	
Grade 2	11 (13)	4 (9)	7 (17)		0 (0)	11 (18)	
Grade 3	1 (1)	0 (0)	1 (2)		0 (0)	1 (2)	
Grade 4	1 (1)	1 (2)	0 (0)		0 (0)	1 (2)	
Time to CRS, days, median (range)	1 (0–13)	1 (0–13)	1 (0–9)	0.12^b^	1 (0–13)	1 (0–11)	0.19^b^
ICANS, *n* (%)							
Total	7 (8)	2 (4)	5 (12)	0.21^b^	3 (12)	4 (6)	0.65^b^
Grade 1	2 (2)	1 (2)	1 (2)		1 (4)	1 (2)	
Grade 2	3 (3)	0 (0)	3 (7)		2 (8)	1 (2)	
Grade 3	0 (0)	0 (0)	0 (0)		0 (0)	0 (0)	
Grade 4	2 (2)	1 (2)	1 (2)		0 (0)	2 (3)	
Time to ICANS, days, median (range)	3 (1–24)	1 (1–1)	7 (1–24)	0.24^b^	7 (3–8)	1 (1–24)	0.40^b^
Admission to ICU, *n* (%)	5 (6)	3 (7)	2 (5)	0.99^a^	1 (4)	4 (6)	0.99^a^
Hospitalization time, days, median (range)	12 (6–86)	12 (6–53)	12.5 (8–86)	0.83^b^	12 (8–86)	13 (6–53)	0.47^b^
IL‐6 peak, pg/mL, median (range)	1208 (15–50000)	938 (15–50000)	1362 (23–50000)	0.13^b^	1208 (16–20156)	1223 (15–50000)	0.79^b^
Ferritin peak, μg/L, median (range)	1060 (81–101815)	903 (81–10303)	1311 (89–101815)	0.33^b^	971 (81–101815)	1076 (114–23676)	0.60^b^
Tocilizumab injection	74 (85)	36 (80)	38 (90)	0.23^a^	22 (88)	52 (84)	0.75^a^
CAR *T* plasma peak, copies/μg cfDNA, median (range)	25,048 (0–757927)^c^	22,737 (0–603028)^c^	25,444 (29–757927)	0.46^b^	22,773 (29–757927)	26,529 (0–603028)^c^	0.54^b^
Time to CAR *T* peak, days, median (range)	13 (1–89)^c^	12 (5–89)^c^	14 (1–38)	0.51^b^	15 (1–35)	13 (5–89)^c^	0.19^b^
CAR *T* at 6M, copies/μg cfDNA, median (range)	97 (0–69930)^c^	97 (0–24143)^c^	91.5 (0–69930)	0.80^b^	64 (0–10789)	99 (0–69930)^c^	0.50^b^
Best response after CAR *T*, *n* (%)^d^							
sCR	41 (49)	17 (39)	24 (60)	0.028^b^	17 (68)	24 (41)	0.037**^b^**
CR	20 (24)	12 (27)	8 (20)		4 (16)	16 (27)	
VGPR	4 (5)	2 (5)	2 (5)		0 (0)	4 (7)	
PR	4 (5)	2 (5)	2 (5)		0 (0)	4 (7)	
SD	4 (5)	1 (2)	3 (8)		2 (8)	2 (3)	
PD	11 (13)	10 (23)	1 (3)		2 (8)	9 (15)	
Relapse, *n* (%)	41 (47)	24 (53)	17 (40)	0.28^a^	9 (36)	32 (52)	0.24^a^
Death, *n* (%)	33 (38)	20 (44)	13 (31)	0.27^a^	7 (28)	26 (42)	0.33^a^
PFS, months, median (range)	7 (0.5–54)	5 (0.5–31)	8 (1–54)	0.034^b^	12 (0.5–54)	6 (0.5–39)	0.008^b^
OS, months, median (range)	12 (0.5–55)	12 (0.5–35)	12.5 (1–55)	0.06^b^	17 (2–54)	9 (0.5–55)	0.023^b^

*Note:* Formatting for *p*‐values < 0.05.

Abbreviations: CAR T, chimeric antigen receptor T‐cell therapy; cfDNA, cell‐free DNA; CR, complete response; CRP, C‐reactive protein; CRS, cytokine release syndrome; ICANS, immune effector cell‐associated neurotoxicity syndrome; ICU, intensive care unit; IL‐6, interleukin 6; OS, overall survival; PD, progressive disease; PFS, progression free survival; PR, partial response; sCR, stringent complete response; SD, stable disease; VGPR, very good partial response.

^a^ Fisher's exact test.

^b^ Mann‐Whitney test.

^c^ 1 is missing.

^d^ 3 are missing.

The A17hom and T17Ahet group had significantly deeper responses to CAR T‐cell therapy (*p* = 0.028), with 24 (60%) versus 17 patients (39%) achieving stringent complete response (sCR) and only 1 patient (3%) in the A17hom and T17Ahet group versus 10 (23%) in the T17hom group with progressive disease (PD). The T17hom cohort also demonstrated higher rates of relapse (53 vs. 40%, *p* = 0.28) and mortality (44 vs. 31%, *p* = 0.27) in comparison to the A17hom and T17Ahet group. PFS differed significantly between the groups (*p* = 0.050) (Figure 2), with a higher median PFS of 8 months in the A17hom and T17Ahet group versus 5 months in the T17hom group (*p* = 0.034). OS differed significantly between the genotype groups (*p* = 0.034). Although the median OS was approximately 0.5 months shorter in the T17hom cohort, this difference did not reach statistical significance (*p* = 0.06). To account for potential bias related to differences in CAR T‐cell product types, treatment outcomes were evaluated specifically in the subset of 52 patients treated with ide‐cel. Patients from the T17hom ide‐cel only cohort showed a shorter PFS (*p* = 0.092) and a significantly decreased OS (*p* = 0.004) compared with the A17hom and T17Ahet group. To exclude potential confounding by ISS stage, patients were stratified according to ISS stage. Within the stratified cohorts of ISS stages I, II and ISS stages II, III, patients from the A17hom and T17Ahet group demonstrated prolonged PFS and OS compared with the T17hom group (Supporting Information S1: Figure 1 and 2).

**FIGURE 2:**
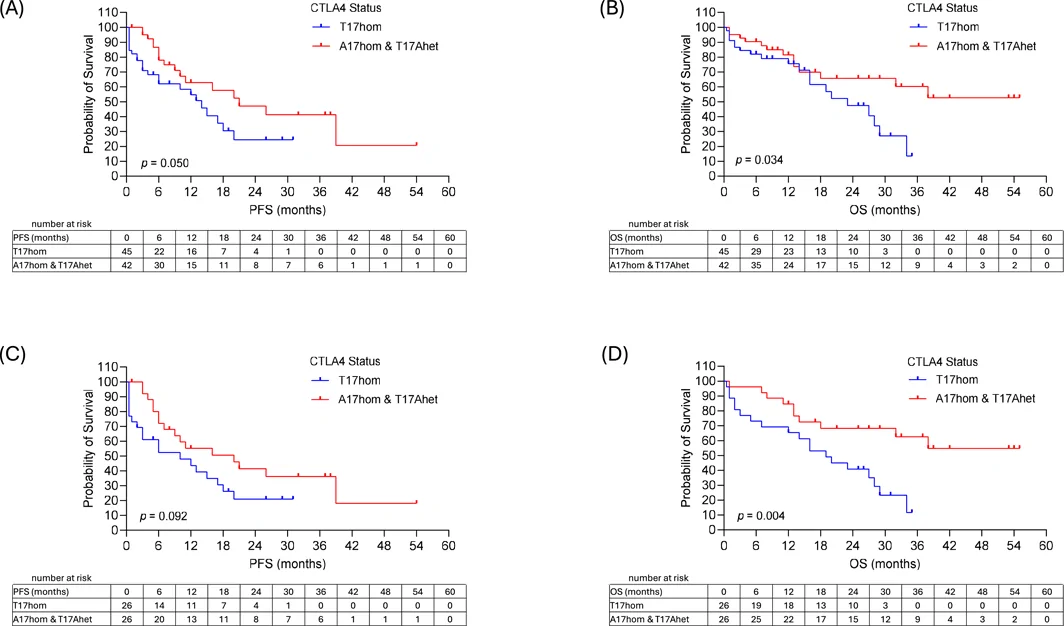
Clinical outcomes in Multiple Myeloma patients with anti‐BCMA CAR T‐cell therapies. Clinical outcomes in the two subgroups CTLA4 T17hom (blue) and A17hom and T17Ahet (red) were analyzed in (A) PFS and (B) OS. Clinical outcomes in patients with ide‐cel CAR T‐cell therapy in (C) PFS and (D) OS.

### 3.7 Treatment Outcome Correlation With LAG3 Germline Variant, Univariate Analysis

Clinical outcome following CAR T‐cell therapy was evaluated in 87 patients stratified by their LAG3 genotype (Table 3). CRS was significantly more frequent and higher grade in patients carrying the I455hom and I455Thet genotype than the T455hom genotype (89 vs. 80%, *p* = 0.024). In line with this observation, the median peak serum ferritin level was higher in the I455hom and I455Thet group (1076 vs. 971 μg/L, *p* = 0.60). The incidence of ICANS was higher in the T445hom cohort (12 vs. 6%, *p* = 0.65), with a delayed median onset compared with the I455hom and I455Thet group (7 vs. 1 day, *p* = 0.40). Rates of intensive care unit admission and duration of hospitalization were comparable between the two groups. CfDNA was used as surrogate for CAR T‐cell expansion. Patients in the T455hom cohort exhibited a lower peak cfDNA CAR T‐cell expansion in plasma (22,773 vs. 26,529 copies/μg cfDNA, *p* = 0.54) and reduced cfDNA CAR T‐cell persistence at 6 months (64 vs. 99 copies/μg cfDNA, *p* = 0.50). This result appears counterintuitive, but possibly cfDNA levels may not directly reflect functional CAR T‐cell activity and viability.

The T455hom group demonstrated significantly superior responses to CAR T‐cell therapy (*p* = 0.037), with 17 patients (68%) achieving sCR and only 2 (8%) in PD, compared to 24 patients (41%) in sCR and 9 patients (15%) in PD in the I455hom and I455Thet group. Furthermore, patients with the T455hom genotype showed lower relapse rates (36 vs. 52%, *p* = 0.24) and reduced mortality (28 vs. 42%, *p* = 0.33). PFS differed significantly between the two cohorts (*p* = 0.045) (Figure 3), with a shorter median PFS of 6 months in the I455hom and I455Thet group compared to 12 months in the T455hom group (*p* = 0.008). In addition, OS was inferior in the I455hom and I455Thet group (*p* = 0.070), with a median OS of 9 versus 17 months in the T455hom cohort (*p* = 0.023). To mitigate potential bias arising from differences in CAR T‐cell product types, treatment outcomes were further analyzed in a subset of 52 patients who received ide‐cel. Within this subgroup, patients carrying the T455hom genotype demonstrated a superior PFS (*p* = 0.055) and OS (*p* = 0.063) compared to those in the I455hom and I455Thet genotype group. To address potential confounding by ISS stages, patients were stratified by ISS. In both the ISS I, II and ISS II, III subgroups, patients harboring the T455hom genotype exhibited longer PFS and OS compared with the I455hom and I455Thet group (Supporting Information S1: Figure 2 and 3).

**FIGURE 3:**
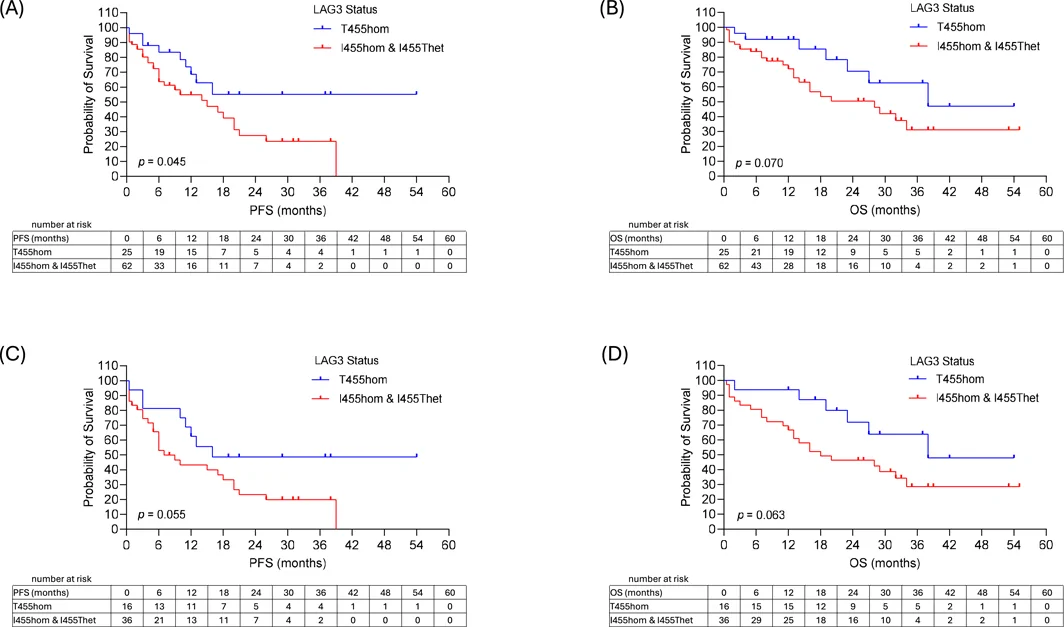
Clinical outcomes in Multiple Myeloma patients with anti‐BCMA CAR T‐cell therapies. Clinical outcomes in the two subgroups LAG3 T455hom (blue) and I455hom and I455Thet (red) were analyzed in (A) PFS and (B) OS. Clinical outcomes in patients with ide‐cel CAR T‐cell therapy in (C) PFS and (D) OS.

### 3.8 Treatment Outcome Correlation With CTLA4 and LAG3 Germline Variants, Multivariate Analysis

Univariate and multivariate Cox proportional hazard models were used to evaluate predictors of PFS and OS (Table 4). Schoenfeld Residual Test was applied to test for the assumption of proportional hazards (Supporting Information S1: table 1 and table 2). Complete cases were considered for this analysis (seven patients were excluded for missing ISS). The LAG3 T455hom genotype was significantly associated with an improved PFS (HR 0.48, *p* = 0.028) in both the univariate and the multivariate analysis (HR 48, *p* = 0.029. Regarding OS, the univariate analysis showed a trend toward improved OS (HR 0.47, *p* = 0.074) In the univariate analysis, the CTLA4 T17hom genotype showed a trend toward a lower PFS (HR 1.73, *p* = 0.060) and was significantly associated with an inferior OS (HR 2.20, *p* = 0.035). In the multivariate analysis, this effect was less apparent. Analysis of combined CTLA4 and LAG3 genotypes revealed that patients harboring the T17hom genotype together with the I455hom or I455Thet genotype exhibited the shortest PFS (*p* = 0.063) (Supporting Information S1: Figure 3). In contrast, patients with the A17hom or T17Ahet genotype in combination with the T455hom genotype demonstrated the longest OS (*p* = 0.054).

**TABLE 4:** Clinical outcomes after CAR T‐cell therapy, multivariable analysis. Results are based on 33 events in the overall survival model and 52 events in progression free surival.

Predictors	Univariate				Multivariable			
	PFS		OS		PFS		OS	
	HR	*p*‐value	HR	*p*‐value	HR	*p*‐value	HR	*p*‐value
LAG3 T455hom versus I455hom and I455Thet	0.48	0.028	0.47	0.074	0.48	0.029	0.47	0.13
CTLA4 T17hom versus A17hom and T17Ahet	1.73	0.060	2.20	0.035	1.30	0.4	1.40	0.5
Age	0.99	0.7	1.01	0.7	0.98	0.4	1.01	0.7
Male versus female	0.64	0.13	0.65	0.2	0.70	0.3	0.87	0.8
ISS III versus I and II	1.13	0.5	1.05	0.8	1.24	0.3	1.00	> 0.9
Remission status pre CAR *T* versus CR								
VGPR	0.82	0.8	0.64	0.7	0.61	0.6	0.42	0.8
PR	1.36	0.6	1.09	0.9	0.69	0.6	0.56	0.6
SD	0.98	0.9	0.72	0.7	0.78	0.7	0.66	0.7
PD	1.53	0.5	1.26	0.8	1.15	0.8	0.81	0.8
Chemo‐tx lines < 3 versus > 3	0.62	0.10	0.78	0.5	0.83	0.6	1.11	0.8
Cilta‐cel versus ide‐cel	0.52	0.087	0.54	0.3	0.53	0.2	0.52	0.3
Extramedullary disease	0.75	0.5	1.78	0.2	1.47	0.5	3.34	0.053
CRP (mg/l)	1.01	0.002	1.02	< 0.001	1.01	0.2	1.01	0.032

*Note:* Bold formatting for *p*‐values < 0.05.

Abbreviations: CAR T, chimeric antigen receptor T‐cell therapy; chemo‐tx, chemotherapy; CR, complete response; HR, hazard ratio; ISS, international staging system; OS, overall survival; PD, progressive disease; PFS, progression free survival; PR, partial response; SD, stable disease; VGPR, very good partial response.

Clinical variables as age, sex and ISS stage were not significantly associated with PFS or OS. Similarly, remission status prior to CAR T‐cell therapy did not demonstrate a significant association. Patients receiving fewer than three prior lines of chemotherapy showed a trend toward improved PFS (HR 0.62, *p* = 0.10) in univariate analysis. This was not maintained in the multivariate analysis. Regarding CAR T‐cell product, treatment with cilta‐cel compared with ide‐cel demonstrated a trend toward improved PFS (HR 0.52, *p* = 0.087) in the univariate analysis as well as toward superior PFS (HR 0.48, *p* = 0.086) and OS (HR 0.48, *p* = 0.086) in the multivariate analysis. To assess the influence of high‐risk cytogenetics, which were available in only a subset of patients, we performed a sensitivity analysis restricted to patients with available cytogenetic data (Supporting Information S1: Table 3). In this sensitivity analysis, high‐risk cytogenetics were associated with statistically worse PFS and a trend for worse OS. However, it should be noted, that the number in the subgroups is small, and the limited number of events may increase the risk of overfitting. Therefore, the analysis must be interpreted with caution.

## 4 Discussion

In this retrospective study, we investigated the prevalence of the CTLA4 rs231775 and LAG3 rs870849 alleles and their association with clinical outcomes in RRMM patients treated with anti‐BCMA CAR T‐cell therapy. Among the 87 patients analyzed, CTLA4 rs231775 exhibited a MAF of 0.29, which is remarkably lower than the reported MAF of 0.37 in the European population (ALFA sample size 509,524, https://www.ncbi.nlm.nih.gov/snp, accessed on November 18, 2025). Consistent with our findings, previous studies have likewise reported a reduced MAF of CTLA4 rs231775 in cohorts of patients with MM and DLBCL [11, 22] Furthermore, another study suggested that this CTLA4 SNP may exert a protective effect against cancer, thereby supporting the observation of a lower MAF in cancer patient populations [23]. In line with this notion, patients harboring the T17hom genotype demonstrated a higher ISS score at initial diagnosis and a higher prevalence of high‐risk cytogenetic abnormalities compared with patients carrying the minor allele.

In contrast, LAG3 rs870849 displayed a significantly higher MAF of 0.58 in our patient cohort compared with the European reference population (0.39) (ALFA sample size 588,090, https://www.ncbi.nlm.nih.gov/snp, accessed on November 18, 2025). An increased MAF of LAG3 rs870849 in patients with hematologic malignancies has been reported in previous studies. Collectively, these findings support the hypothesis that LAG3 rs870849 may contribute to cancer susceptibility [17, 20, 22].

The CTLA4 rs231775 allele may represent a prognostic factor in patients with MM. Previous studies have shown that polymorphism, specifically the rs231775 SNP, results in reduced upregulation of CTLA‐4 expression following T‐cell activation and, consequently, enhanced T‐cell proliferative capacity [24]. Consistent with these functional observations, our study demonstrated a significantly improved clinical outcome in patients harboring the A17hom or T17Ahet genotypes compared with T17hom patients. The A17hom and T17Ahet group exhibited a median PFS of 8 months, compared to 5 months in the T17hom group. These findings are consistent with previously reported data in cohorts of patients with DLBCL treated with CAR T‐cell therapy [11]. Supporting the concept of reduced CTLA‐4 signaling in patients harboring A17hom or T17Ahet, this genotype group exhibited a higher incidence of CRS, suggesting enhanced immune cell activation and increased CAR T‐cell activity.

LAG3 rs870849, like CTLA4 rs231775, may similarly serve as a prognostic factor in MM patients. Following CAR T‐cell therapy, carriers of the T455hom genotype exhibited significantly higher ORRs, as well as superior PFS and OS compared with I455hom and I455Thet patients. Favorable outcomes in T455hom carriers have also been observed in cohorts of DLBCL patients and in MM patients treated with ASCT [17, 20] Mechanistically, LAG‐3 inhibitory function is regulated by dimerization, ligand binding, and metalloprotease‐mediated cleavage, with disruption of these processes enhancing T‐cell proliferation and antitumor activity, as well as improving the therapeutic benefit of tumor specific cellular products. This aligns with the concept that genetic or structural downregulation of LAG‐3 may confer enhanced clinical benefit [25, 26, 27]. By contrast, in patients with various hematological diseases who underwent allogeneic hematopoietic stem cell transplantation, the presence of donor T455 alleles was linked to inferior OS, proposing that reduced LAG‐3 inhibition can be beneficial in cancer therapy but detrimental in hematopoietic transplantation [28]. Furthermore, the T455hom subgroup exhibited a significantly lower incidence and reduced severity of CRS compared with the I455hom and I455Thet genotype group, which contrasts with the expectation of enhanced immune cell activity in T455hom. In a separate study of patients with DLBCL, the T455hom subgroup demonstrated a higher incidence of CRS [17]. These discrepancies may reflect disease‐ or context‐specific effects or could be influenced by the limited cohort size in this study.

In the present cohort, analysis of hazard ratios revealed that LAG3 and CTLA4 genotypes were significantly associated with clinical outcomes. In contrast, other variables, including sex, age, ISS stage, CAR T‐cell product, and remission status prior to therapy showed no significant correlation.

It should be noted that these findings are based on a single‐center cohort of 87 patients, limiting statistical power. Prior studies in recipients of BCMA‐directed CAR T‐cell therapies have identified previous stem cell transplantation, light‐chain MM, and high‐risk cytogenetics as adverse prognostic factors, emphasizing the need for validation in larger, multi‐center cohorts to minimize potential bias [29, 30] In addition, previous studies have reported superior efficacy of cilta‐cel compared with ide‐cel [31, 32] To account for this potential confounding factor, the cohort was stratified by CAR T‐cell product, revealing that the observed genotype associated effects persisted within the ide‐cel treated patient subgroup. Given that cilta‐cel is a more recently approved therapy, the shorter follow‐up duration limits the ability to detect statistically significant differences between the genotypes (Supporting Information S1: Figure 4).

Regarding toxicities, the CTLA4‐associated toxicity pattern, which is broadly consistent with enhanced immune activation in patients with superior clinical outcome, the LAG3 findings are less clear: The T455hom subgroup demonstrated improved clinical efficacy despite a lower frequency of CRS. While the underlying mechanism of action remains unclear, this result may suggest that treatment efficacy is not solely dependent on inflammatory activity, but also on differences in CAR T‐cell functional quality and viability and other immune regulations.Despite the limitations ofarticle, mainly the retrospective design and the small sample size, we observed robust and consistent associations between LAG3 and CTLA4 genotypes and key clinical outcomes. These findings allow to discuss the hypothesis of a contributory role of germline immune checkpoint variants in shaping CAR T‐cell efficacy and highlight CTLA4 and LAG3 as predictive biomarkers and potential therapeutic targets. If the results could be confirmed in prospective larger cohorts, this mechanism could provide a biological rationale for combining CAR T‐cell therapy with immune checkpoint inhibitors. This approach has already demonstrated improved response rates, PFS and OS due to combination of CAR T‐cell therapy with PD‐1 blockade across lymphomas and multiple solid malignancies [33]. Given the ongoing clinical development of multiple CTLA‐4 and LAG‐3 antibodies, these checkpoints represent promising candidates for combination strategies to enhance CAR T‐cell efficacy [34, 35] Importantly, preclinical studies provide mechanistic support for this concept. Genetic disruption of CTLA4 or LAG3 in CAR T‐cells augments antitumor efficacy by sustaining CD28‐mediated signaling, thereby improving effector persistence and mitigating T‐cell exhaustion [10, 25].

## 5 Conclusions

The CTLA4 rs231775 and LAG3 rs870849 allele may have a significant impact on clinical outcomes following CAR T‐cell therapy in RRMM patients. Notably, with all the limitations of a retrospective analysis, the presence of the minor alleles of both variants was associated with improved clinical outcomes in our study. Carriers of the CTLA4 rs231775 minor allele exhibited significantly longer PFS and OS. Similarly, patients homozygous for the minor allele of LAG3 rs870849 demonstrated a significantly longer PFS and a trend toward improved OS compared with individuals carrying the major allele.

These findings suggest to further evaluate the CTLA4 and LAG3 polymorphisms for their potential to serve as biomarkers for risk classification and provide a biological rationale for therapeutic strategies aimed at reducing CTLA‐4 and LAG‐3 signaling to enhance CAR T‐cell efficacy. Independent studies should further evaluate and confirm these data.

## Author Contributions

**Elisa Suter:** formal analysis, investigation, writing – original draft preparation, visualization. **Martina Bertschinger:** formal analysis, investigation, resources, writing – original draft preparation. **Inna Shaforostova:** resources, writing – review and editing. **Marie‐Noelle Kronig:** resources, writing – review and editing. **Henning Nilius:** formal analysis, writing – review and editing. **Ulrike Bacher:** resources, writing – review and editing. **Katja Seipel:** conceptualization, formal analysis, investigation, writing – review and editing. **Thomas Pabst:** conceptualization, supervision, project administration. All authors have read and agreed to the published version of the manuscript.

## Funding

The authors have nothing to report.

## Ethics Statement

The study was conducted according to the guidelines of the Declaration of Helsinki and approved by decisions of the local ethics committee of Bern, Switzerland, decision number 2025‐00853, date of approval April 24, 2025.

## Conflicts of Interest

The authors declare no conflicts of interest.

## Supporting information


Supporting Information S1

## Data Availability

The data that support the findings of this study are available from the corresponding author upon reasonable request.
