# Supplementary material for: Clinical Impact of CTLA4 and LAG3 Single‐Nucleotide Polymorphisms in Multiple Myeloma Patients Treated With BCMA CAR T‐Cell Therapy
Source: Hematol Oncol. 2026 Sep 28;44(6):e70267. doi: 10.1002/hon.70267 (PMC13620279; doi:10.1002/hon.70267)
Supplement: Supplementary file 1 [file HON-44-e70267-s001.docx]

### Supplemental Figures

#### Supplemental Figure 1

#### Supplemental Figure 2

#### Supplemental Figure 3

#### Supplemental Figure 4

### Supplemental Figure Legends

Supplemental Figure 1. Clinical outcomes in Multiple Myeloma patients with anti-BCMA CAR-T cell therapy with initial ISS stages of I or II. Clinical outcomes in the two subgroups CTLA4 T17hom (blue) and A17hom & T17Ahet (red) were analyzed in (A) PFS and (B) OS. Clinical outcomes in the two subgroups LAG3 T455hom (blue) and I455hom & I455Thet (red) were analyzed in (C) PFS and (D) OS.

Supplemental Figure 2. Clinical outcomes in Multiple Myeloma patients with anti-BCMA CAR-T cell therapy with initial ISS stages of II or III. Clinical outcomes in the two subgroups CTLA4 T17hom (blue) and A17hom & T17Ahet (red) were analyzed in (A) PFS and (B) OS. Clinical outcomes in the two subgroups LAG3 T455hom (blue) and I455hom & I455Thet (red) were analyzed in (C) PFS and (D) OS.

Supplemental Figure 3. Clinical outcomes in Multiple Myeloma patients with anti-BCMA CAR-T cell therapy according to their CTLA4 and LAG3 genetic variants combinations. Clinical outcomes in the four subgroups T17hom - T455hom (blue), T17hom - I455hom & I455Thet (red), A17hom & T17Ahet - T455hom (green) and A17hom & T17Ahet - I455hom & I455Thet (violet) in (A) PFS and (B) OS.

Supplemental Figure 4. Clinical outcomes in Multiple Myeloma patients with cilta-cel CAR-T cell therapy. Clinical outcomes in the two subgroups CTLA4 T17hom (blue) and A17hom & T17Ahet (red) were analyzed in (A) PFS and (B) OS. Clinical outcomes in the two subgroups LAG3 T455hom (blue) and I455hom & I455Thet (red) were analyzed in (C) PFS and (D) OS.

### Supplemental Tables

**TableS1**: Results of the Schoenfeld residuals test (cox.zph) assessing the proportional hazards assumption for the Cox regression model of overall survival.

| Variable | Chi-sq | df | p |
| --- | --- | --- | --- |
| LAG3 T455hom vs I455hom & I455Thet | 0.66444 | 1 | 0.415 |
| CTLA4 T17hom vs A17hom & T17Ahet | 3.95569 | 1 | 0.047 |
| Age | 0.74516 | 1 | 0.388 |
| Male vs female | 0.36067 | 1 | 0.548 |
| ISS III vs I&II | 0.00427 | 1 | 0.948 |
| Remission status pre CAR T vs CR | 2.30019 | 4 | 0.681 |
| Chemo-tx lines <3 vs >3 | 0.93347 | 1 | 0.334 |
| Cilta-cel vs ide-cel | 0.75741 | 2 | 0.685 |
| Extramedullar disease | 0.03185 | 1 | 0.858 |
| CRP | 0.14514 | 1 | 0.703 |
| GLOBAL | 10.50892 | 14 | 0.724 |

ISS: international staging system; CAR T: chimeric antigen receptor T-cell therapy

| Variable | Chi-sq | df | p |
| --- | --- | --- | --- |
| LAG3 T455hom vs I455hom & I455Thet | 0.01118 | 1 | 0.916 |
| CTLA4 T17hom vs A17hom & T17Ahet | 0.00922 | 1 | 0.924 |
| Age | 0.44094 | 1 | 0.507 |
| Male vs female | 2.24259 | 1 | 0.134 |
| ISS III vs I&II | 0.00652 | 1 | 0.936 |
| Remission status pre CAR T vs CR | 3.54369 | 4 | 0.471 |
| Chemo-tx lines <3 vs >3 | 1.34093 | 1 | 0.247 |
| Cilta-cel vs ide-cel | 2.09613 | 2 | 0.351 |
| Extramedullar disease | 3.00083 | 1 | 0.083 |
| CRP | 0.18767 | 1 | 0.665 |
| GLOBAL | 10.00084 | 14 | 0.762 |

**TableS2**: Results of the Schoenfeld residuals test (cox.zph) assessing the proportional hazards assumption for the Cox regression model of the progression free survival.

ISS: international staging system; CAR T: chimeric antigen receptor T-cell therapy

**Table S3.** Clinical outcomes after CAR T-cell therapy, multivariable analysis. Results are based on 33 events in the Overall Survival Model and 52 events in Progression Free Survival. Based on 38 patients

| Predictors | Univariate | | | | Multivariable | | | |
| --- | --- | --- | --- | --- | --- | --- | --- | --- |
|  | PFS | | OS | | PFS | | OS | |
|  | HR | *p*-value | HR | *p*-value | HR | *p*-value | HR | *p*-value |
| LAG3 T455hom vs I455hom & I455Thet | 0.48 | **0.028** | 0.47 | 0.074 | 0.58 | **0.4** | 0.60 | 0.5 |
| CTLA4 T17hom vs A17hom & T17Ahet | 1.73 | 0.060 | 2.20 | **0.035** | 0.49 | .3 | 2.04 | 0.4 |
| Age | 0.99 | 0.7 | 1.01 | 0.7 | 0.99 | 0.9 | 1.01 | 0.7 |
| Male vs female | 0.64 | 0.13 | 0.65 | 0.2 | 0.37 | 0.084 | 0.43 | 0.82 |
| ISS III vs I&II | 1.13 | 0.5 | 1.05 | 0.8 | 0.24 | 0.011 | 0.28 | 0.082 |
| Remission status pre CAR T vs CR | | | | | | | | |
| VGPR | 0.82 | 0.8 | 0.64 | 0.7 | 0.35 | 0.5 | 0.67 | 0.8 |
| PR | 1.36 | 0.6 | 1.09 | 0.9 | 2.46 | 0.4 | 1.50 | 0.8 |
| SD | 0.98 | 0.9 | 0.72 | 0.7 | 0.25 | 0.2 | 0.42 | 0.6 |
| PD | 1.53 | 0.5 | 1.26 | 0.8 | 2.03 | 0.5 | 0.43 | 0.6 |
| Chemo-tx lines <3 vs >3 | 0.62 | 0.10 | 0.78 | 0.5 | 0.58 | 0.5 | 0.73 | 0.8 |
| Cilta-cel vs ide-cel | 0.52 | 0.087 | 0.54 | 0.3 | 0.4 | 0.3 | 0.47 | 0.6 |
| Extramedullar disease | 0.75 | 0.5 | 1.78 | 0.2 | 23.3 | 0.005 | 68.2 | 0.010 |
| CRP (per mg/l) | 1.01 | **0.002** | 1.02 | **< 0.001** | 0.99 | 0.4 | 1.00 | **>0.9** |
| High-Risk Cytogenetics (2024) | 1.72 | **0.2** | 2.15 | **0.14** | 9.02 | 0.009 | 5.16 | **0.12** |

PFS: progression free survival; OS: overall survival; HR: hazard ratio; ISS: international staging system; CAR T: chimeric antigen receptor T-cell therapy; CR: complete response; VGPR: very good partial response; PR: partial response; SD: stable disease; PD: progressive disease; chemo-tx: chemotherapy. Bold formatting for *p*-values < 0.05.
